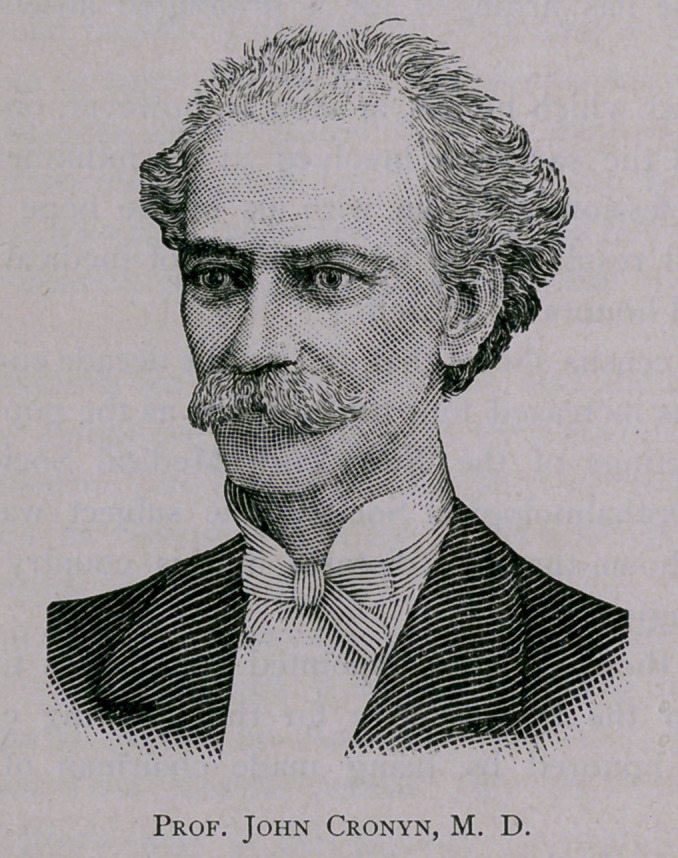# The New York State Medical Association

**Published:** 1887-12

**Authors:** 


### *THE NEW YORK STATE MEDICAL ASSOCIATION.*

This Association held its fourth annual meeting in New York late in September. Owing to the great interest taken in the International Medical Congress, and the amount of space taken

PROF. JOHN CRONYN, M. D.

by its reports in this journal, the notices of the meeting were omitted. The Association was well attended, and many distinguished foreign physicians remained to attend its sessions. The articles and addresses presented were said to excel any offered in the past. The address of the second day was given by Prof. John Cronyn, of the Medical Department of Niagara University, of this city. The Association closed its labors by the election of Dr. Cronyn as President. This is a most fitting tribute to Dr. Cronyn, who is recognized as one of the foremost physicians of Western New York. His labors in behalf of higher medical education are well known to all our readers. He is a distinguished teacher, an eminent citizen, and occupies a high position in this community, where he has practiced for thirty-odd years.

We are able to present our readers a picture of the Doctor, by the courtesy of the Buffalo *Morning Express.*

---

AMONG the awards to exhibitors at the American exhibition, London, was a gold medal to Fairchild Bros. & Foster, New York, for " digestive ferments," extractum pancreatis, peptonizing powders, and pepsine in scales. All who have used these elegant, reliable and convenient preparations for peptonizing food, will recognize that this recognition of their merit is well deserved.

---

THE JOURNAL, as well as our readers, are under obligations to Dr. W. W. Potter for many valuable editorials received from time to time. The Doctor is a clear and concise writer.

## Figures and Tables

**Figure f1:**